# Emergence and Prevalence of *Vibrio cholerae* O1 Sequence Type 75 Clonal Complex, Fujian Province, China, 2009–2023

**DOI:** 10.3201/eid3107.241838

**Published:** 2025-07

**Authors:** Zili Ke, Bo Pang, Jinsong Yang, Yadong Gao, Xiaoxuan Zhang, Haibin Xu, Chaochen Luo, Biao Kan

**Affiliations:** Fujian Center for Disease Control and Prevention, Fuzhou, China (Z. Ke, J. Yang, Y. Gao, H. Xu, C. Luo); National Institute for Communicable Disease and Prevention, Chinese Center for Disease Control and Prevention, Beijing, China (B. Pang, B. Kan); Fujian Provincial Key Laboratory of Zoonosis Research, Fuzhou (J. Yang); School of Public Health, Fujian Medical University, Fuzhou (J. Yang, X. Zhang)

**Keywords:** Cholera, Vibrio cholerae, bacteria, antimicrobial resistance, enteric infections, genomics, sequence type 75, China

## Abstract

We investigated the molecular epidemiology of *Vibrio cholerae* O1 in Fujian Province, China, during 2009­–2023. Sequence type (ST) 75 clonal complex has emerged continuously since 2020, and ST1480 and ST182 have dominated. ST1480 strains appear to have widespread transmission. To monitor *V. cholerae* evolution, continued global genomic surveillance is needed.

Cholera, an acute and severe diarrheal disease, remains a major public health problem in developing countries. The *Vibrio cholerae* O1 El Tor strain is responsible for the ongoing seventh pandemic of cholera (7PC) (*1*). Most 7PC clinical isolates have belonged to sequence type (ST) 69 (*2*–*4*). However, in the 2010s, ST75 instead of ST69 has emerged and become the dominant clonal group in China and South Africa, and it has several derived populations, including ST169, ST170, and ST182 (*5*,*6*).

Although Fujian, China, experienced many 7PC outbreaks in the past 100 years, cholera cases caused by ST69 strains decreased dramatically after 2006. However, diarrheal diseases caused by *V. cholerae* ST75 and its derivatives have been reported continuously during 2018–2023. To determine the molecular epidemiology of *V. cholerae* in Fujian Province, especially among ST75 and its derivatives, we performed a whole-genome sequence–based study of *V. cholerae* O1 isolated from clinical and environment samples during 2009–2023. The Medical Ethics Committee of Fujian Center for Disease Control and Prevention provided ethical approval for this study.

## The Study

During 2009–2023, a total of 20 cases of diarrhea caused by *V. cholerae* O1 were reported in Fujian Province. We selected 1 *V. cholerae* isolate from each patient for this study and removed duplicate patient isolates (Table). All 20 isolates were serotype Ogawa, except 2 serotyped as Inaba. The epidemiologic data showed that all the patients were from China and had never traveled abroad. Twelve patients were male and 8 female, a ratio of 1.5:1. One (5.0%) patient was <14 years of age, and the other 19 (95.0%) were 15–65 years of age. During 2009–2019, only 6 sporadic cases were reported, but 1 outbreak (n = 2 cases) occurred in 2021 and 2 outbreaks (n = 3 cases per outbreak) occurred in 2023. 

**Table:** Distribution of sequence types from clinical isolates in a study of emergence and prevalence of *Vibrio cholerae* O1 ST75 clonal complex, Fujian Province, China, 2009–2023*

Category	ST	2009	2011	2012	2019	2020	2021	2022	2023	Total
ST75 and derivatives	75	0	0	2	0	0	0	1	0	3
	1480	0	0	0	0	1	2	0	3	6
	182	0	0	0	0	0	4	0	0	4
	170	0	0	0	0	0	0	0	3	3
	169	0	1	0	0	0	0	0	0	1
Others	176	1	0	0	0	0	0	0	0	1
	167	0	0	0	2	0	0	0	0	2

*ST, sequence type.

The results of retrospective survey showed that 75% (15/20) of the patients had eaten seafood within 3 days before illness onset, and the 3 most suspicious foods were shellfish, marine fish, and softshell turtle. All the patients recovered after treatment, and no secondary transmission was observed. 

We studied the 20 isolates by obtaining whole-genomic sequences. We submitted short-read sequence data to the National Center for Biotechnology Information (BioProject accession no. PRJNA1191620). We also provide GenBank accession numbers for global sequences used in this study (Appendix 1). 

Of the 20 isolates, we identified 7 STs by multilocus sequence typing (MLST), including ST75 (n = 3), ST1480 (n = 6), ST182 (n = 4), ST170 (n = 3), ST169 (n = 1), ST176 (n = 1), and ST167 (n = 2) (Table). In the 2 Inaba isolates, we found a previously reported transposase insertion in the *rfbT* gene (*7*), which caused the serotype conversion. 

For population structural analysis, we constructed a minimum spanning tree on the basis of MLST data (Appendix 2 Figure 1). According to the rules of eBURST (*8*), ST75, ST1480, ST182, ST170, and ST169 could be defined as a clonal complex (CC) of ST75. Using 2,443 core genes ubiquitous in *V. cholerae*, we obtained a minimum spanning tree of the 20 isolates from Fujian by using a core-genome MLST scheme (*9*) (Appendix 2 Figure 2). We found that during 2009–2019, ST75 derivatives were relatively dormant, and we only identified ST169 during that timeframe. However, after the appearance of ST1480 in 2020, ST75 derivatives were distinctly active, and ST1480, ST182, and ST170 emerged within 4 years. Compared with other ST75 derivatives, such as ST169 and ST170, the genetic relationships between ST75, ST1480, and ST182 were closer. The core-genome MLST of ST1480 indicated only 1–43 alleles differences between the isolates collected in 2020, 2021, and 2023.

Most ST75 CC isolates were nontoxigenic, except for 2 ST1480 isolates (strain nos. FJ2021001 and FJ2021002) in 2021 and 1 ST75 isolate (strain nos. FJ2022003) in 2022, which were toxigenic and contained CTX phages of the *ctxB3* genotype. All isolates carried *tcpA*, *rtxA*, *hlyA*, *toxR*, and *Vibrio* pathogenicity islands 1 and 2. However, we did not find *Vibrio* seventh pandemic island I or II in those isolates.

Antimicrobial susceptibility testing by broth dilution method showed that all the ST75 CC isolates were susceptible to ampicillin, cephalosporin, chloramphenicol, tetracycline, ciprofloxacin, and azithromycin. The *qnrVC4* gene was the only antimicrobial-resistance determinant found in those isolates. That gene was in the super integron and is associated with fluoroquinolone resistance (*10*). Although we found the *qnrVC4* gene in the 3 ST75 and 3 ST170 isolates, those isolates did not show fluoroquinolone resistance. Whether the *qnrVC4* gene can mediate resistance to fluoroquinolones in ST75 needs long-term monitoring data.

To investigate the potential origin and phylogenetic relationship of clinical ST75 isolates and their derivatives, we further compared those isolates with 5 environmental strains (2 ST169, 2 ST75, and 1 ST1480) collected from Fujian Province during 2009–2023, and to a global collection of 103 ST75 or closely related ST169, ST170, ST182, ST1480, and ST725–728 genomes (Appendix 1). We constructed a maximum-likelihood phylogenetic tree on the whole-genome single-nucleotide polymorphism (SNP) profiles, which revealed 5 distinct lineages: L3 (Gulf Coast), L3b.1, L3b.2, L3b.3, and L3b.4 (Figure). ST1480 and ST182 clustered in L3b.1, and had a maximum pairwise distance of 0–6 SNPs. We found that 3 ST1480 isolates (strain nos. FJ2021001 and FJ2021002 isolated from patients, and strain no. FJ2014078 isolated from turtle farming water) and 1 ST182 isolate from Fujian (strain no. FJ2021008) were genetically close to 2 ST182 isolates from Zhejiang (strain nos. 11–2_S78 and 11–1_S22 isolated in 2011), and to 2 ST75 isolates from Taiwan (strain R16.3429 isolated in 2010 and strain R16.3447 isolated in 2013). Fujian, Zhejiang, and Taiwan are geographically adjacent in the southeastern region of China. Given the geographic proximity and frequent exchanges among those regions, and because *V. cholerae* O1 ST75 has dominated in Zhejiang and Taiwan since 2009 (*5*,*6*), we speculate that the ST75 derivatives in Fujian shared a common ancestor with the isolates from those areas. ST75 seemed to be a valuable marker for those *V. cholerae* lineages, which differed from the 7PC strains. In addition, the persistence of *V. cholerae* O1 ST75 CC, such as ST1480, in the environment and in patients, especially the emergence of toxigenic isolates, suggest that *V. cholerae* ST75 CC should be monitored more closely.

**Figure:**
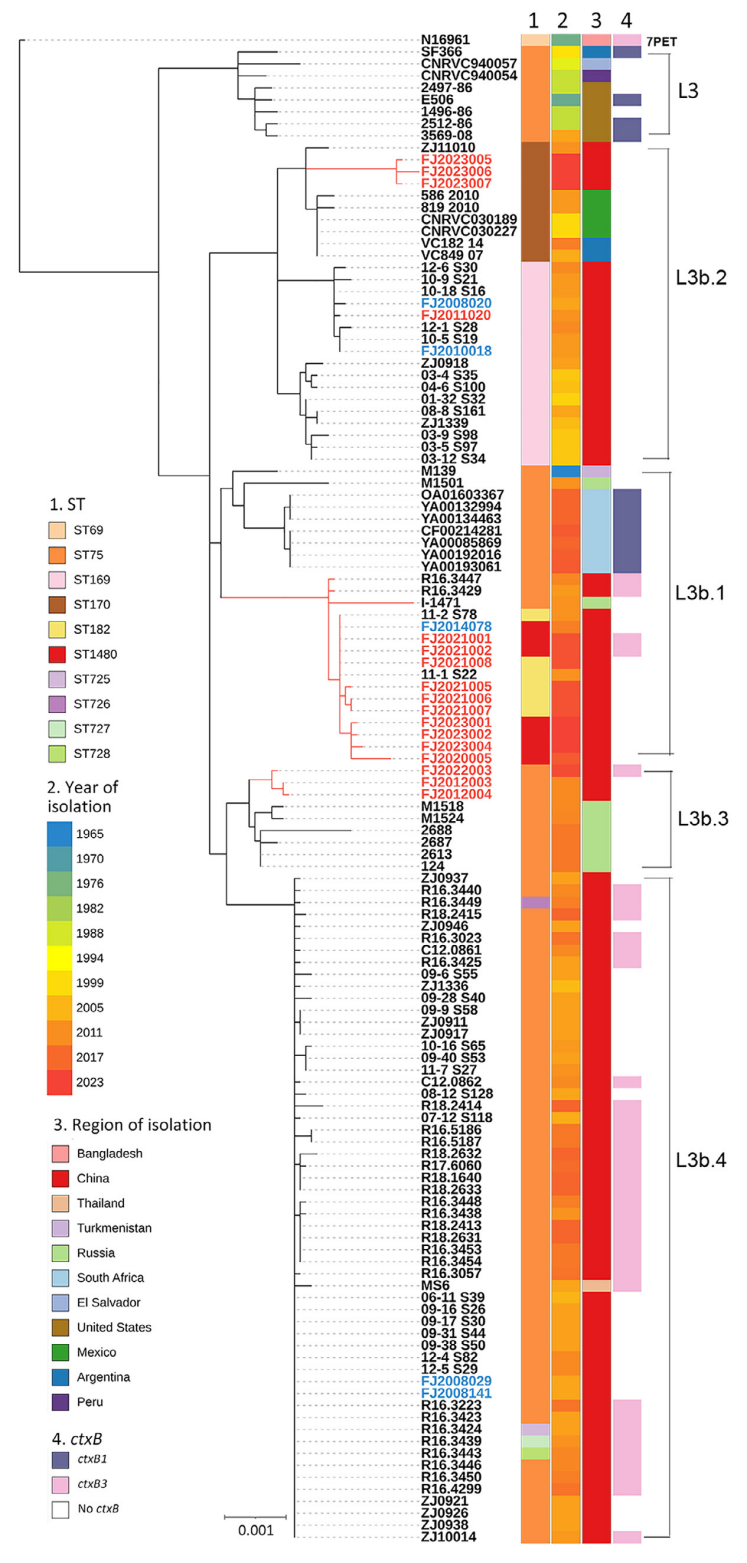
Maximum-likelihood phylogeny of genomic sequences from a study of emergence and prevalence of *Vibrio cholerae* O1 ST75 clonal complex, Fujian Province, China, 2009–2023. The tree revealed 5 distinct lineages: L3 (Gulf Coast), L3b.1, L3b.2, L3b.3, and L3b.4. ST1480 and ST182 clustered in L3b.1 and had a maximum pairwise distance of 0–6 SNPs. The 7PET genome N16961 (ST69) was included as an outgroup. Isolates from Fujian Province are show as red branch lines and labels for clinical isolates (n = 17) and blue text for environmental isolates (n = 5); and the other 102 isolates (black text) are of global origin. Sequence type, year and region of isolation, and types of *ctxB* are shown at the tips of the tree; lineages are marked on the right side of the tree. The scale bar indicates substitutions per variable site. 7PET, 7th pandemic *V. cholerae* O1 El Tor; *ctxB*, cholera toxin B subunit gene; ST, sequence type.

## Conclusions

*V. cholerae* O1 ST75 CC has gradually expanded in Fujian Province, China. The continuous emergence of ST1480, ST182, ST170, and ST169 indicate the dynamic evolution of this CC. Although no cholera epidemic caused by this CC was reported in Fujian before 2021, the frequency of *ctxAB* genes and the toxin coregulated pilus gene cluster in these strains deserves an enhanced whole-genome sequence–based global surveillance to closely monitor their evolution.

Appendix 1 Genomic sequences used a study of emergence and prevalence of *Vibrio cholerae* O1 sequence type 75 clonal complex strains, Fujian Province, China, 2009–2023.

Appendix 2 Additional information on emergence and prevalence of *Vibrio cholerae* O1 sequence type 75 clonal complex strains, Fujian Province, China, 2009–2023.

## References

[1] Kanungo S, Azman AS, Ramamurthy T, Deen J, Dutta S. Cholera. Lancet. 2022;399:1429–40. 10.1016/S0140-6736(22)00330-0 35397865

[2] Narendrakumar L, Jaikumar VS, Chandrika SK, Thomas S. Epidemiological and pathogenic characteristics of Haitian variant *V. cholerae* circulating in India over a decade (2000-2018). Microb Pathog. 2020;149:104538. 10.1016/j.micpath.2020.104538 32987116

[3] Greig DR, Schaefer U, Octavia S, Hunter E, Chattaway MA, Dallman TJ, et al. Evaluation of whole-genome sequencing for identification and typing of *Vibrio cholerae.* J Clin Microbiol. 2018;56:e00831–18. 10.1128/JCM.00831-18 30135231 PMC6204685

[4] Siriphap A, Leekitcharoenphon P, Kaas RS, Theethakaew C, Aarestrup FM, Sutheinkul O, et al. Characterization and genetic variation of *Vibrio cholerae* isolated from clinical and environmental sources in Thailand. PLoS One. 2017;12:e0169324. 10.1371/journal.pone.0169324 28103259 PMC5245877

[5] Smith AM, Weill FX, Njamkepo E, Ngomane HM, Ramalwa N, Sekwadi P, et al. Emergence of *Vibrio cholerae* O1 sequence type 75, South Africa, 2018–2020. Emerg Infect Dis. 2021;27:2927–31. 10.3201/eid2711.211144 34670657 PMC8544974

[6] Luo Y, Octavia S, Jin D, Ye J, Miao Z, Jiang T, et al. US Gulf-like toxigenic O1 *Vibrio cholerae* causing sporadic cholera outbreaks in China. J Infect. 2016;72:564–72. 10.1016/j.jinf.2016.02.005 26920786

[7] Liang W, Wang L, Liang P, Zheng X, Zhou H, Zhang J, et al. Sequence polymorphisms of *rfbT* among the *Vibrio cholerae* O1 strains in the Ogawa and Inaba serotype shifts. BMC Microbiol. 2013;13:173. 10.1186/1471-2180-13-173 23889924 PMC3727987

[8] Spratt BG, Hanage WP, Li B, Aanensen DM, Feil EJ. Displaying the relatedness among isolates of bacterial species — the eBURST approach. FEMS Microbiol Lett. 2004;241:129–34. 10.1016/j.femsle.2004.11.015 15598523

[9] Liang KYH, Orata FD, Islam MT, Nasreen T, Alam M, Tarr CL, et al. A *Vibrio cholerae* core genome multilocus sequence typing scheme to facilitate the epidemiological study of cholera. J Bacteriol. 2020;202:e00086–20. 10.1128/JB.00086-20 32540931 PMC7685551

[10] Fonseca EL, Vicente ACP. Epidemiology of *qnrVC* alleles and emergence out of the Vibrionaceae family. J Med Microbiol. 2013;62:1628–30. 10.1099/jmm.0.062661-0 23800600

