# Supplementary material for: Emergence and Prevalence of Vibrio cholerae O1 Sequence Type 75 Clonal Complex, Fujian Province, China, 2009–2023
Source: Emerg Infect Dis. 2025 Jul;31(7):1428–31. doi: 10.3201/eid3107.241838 (PMC12205453; doi:10.3201/eid3107.241838)
Supplement: Appendix 2 — Additional information on emergence and prevalence of Vibrio cholerae O1 sequence type 75 clonal complex strains, Fujian Province, China, 2009–2023. [file 24-1838-Techapp-s2.pdf]

Article DOI: https://doi.org/10.3201/eid3107.241838

EID cannot ensure accessibility for supplementary materials supplied by authors. Readers who have difficulty accessing supplementary content should contact the authors for assistance.

Emergence and Prevalence of *Vibrio cholerae* O1 Sequence Type 75 Clonal Complex, Fujian Province, China, 2009–2023

Appendix 2

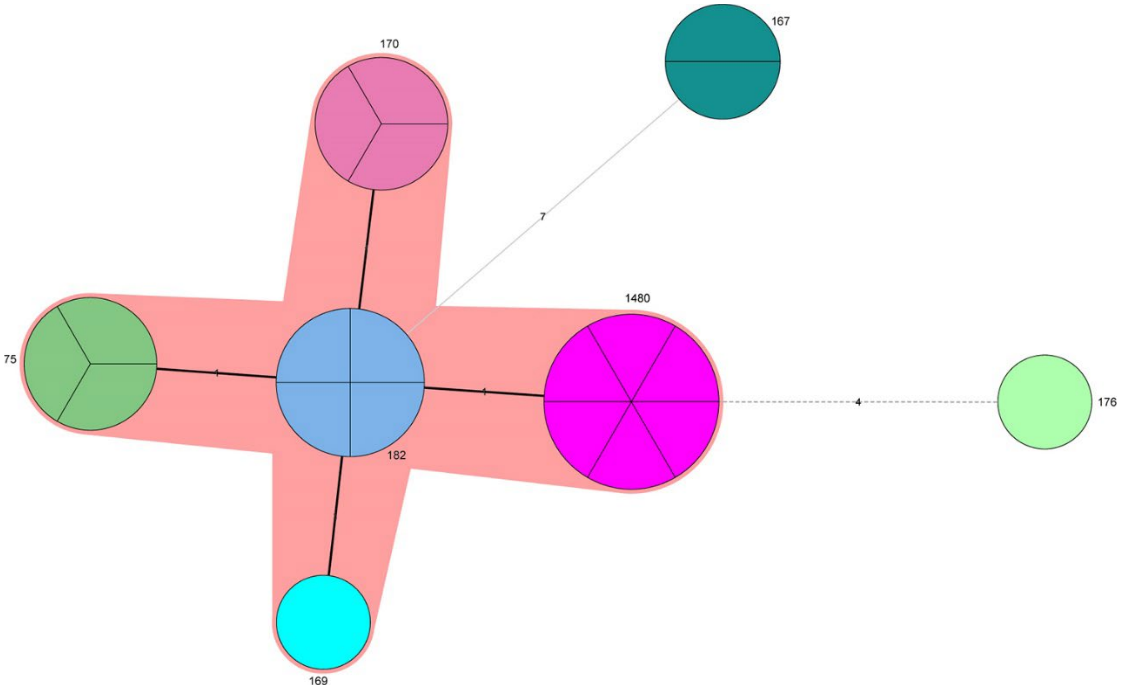

Appendix 2 Figure 1. Minimum spanning trees were constructed according to the ST allele profiles of the 20 *V. cholerae*. Seven ST types of Fujian isolates were labeled with different colors. The genetic distances between the different STs were shown on the branches, and the size of the circle indicated the number of isolates. ST75 CC was defined as a group of STs closely related to ST75, sharing 6 of 7 alleles, and it can be clearly partitioned in pink.

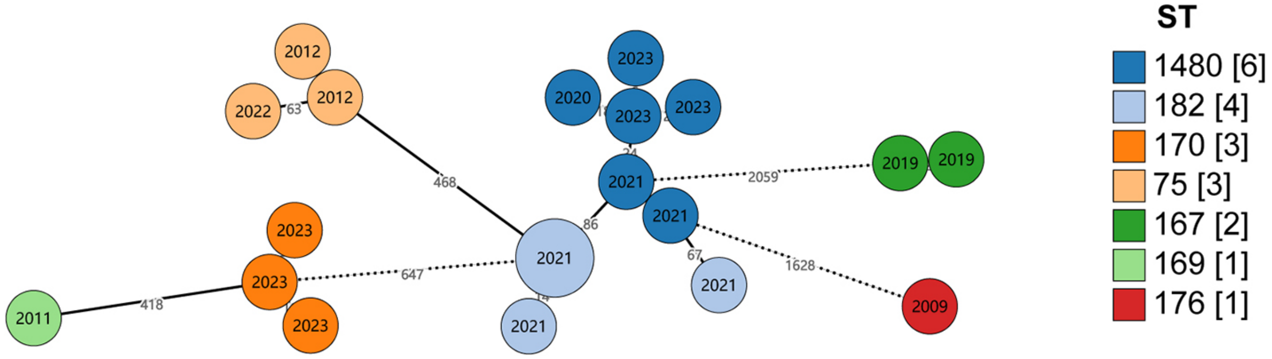

Appendix 2 Figure 2. Minimum spanning trees were constructed according to the core genome allele profiles of 20 *Vibrio cholerae* isolates. The nodes were color-labeled according to the sequence type of the isolates. The genetic distance between different clusters is indicated on the branches, and branches with a length greater than 500 were shortened. The size of the circle indicates the number of isolates.
